# Effectiveness of a resistance training program on physical function, muscle strength, and body composition in community-dwelling older adults receiving home care: a cluster-randomized controlled trial

**DOI:** 10.1186/s11556-020-00243-9

**Published:** 2020-08-07

**Authors:** Hilde Bremseth Bårdstu, Vidar Andersen, Marius Steiro Fimland, Lene Aasdahl, Truls Raastad, Kristoffer T. Cumming, Atle Hole Sæterbakken

**Affiliations:** 1 Department of Sport, Food and Natural Sciences, Faculty of Education, Arts and Sports, Western Norway University of Applied Sciences, PB 133, 6851 Sogndal, Norway; 2 Department of Neuromedicine and Movement Science, Faculty of Medicine and Health Sciences, Norwegian University of Science and Technology, Trondheim, Norway; 3 Unicare Helsefort Rehabilitation Centre, Rissa, Norway; 4 Department of Public Health and Nursing, Faculty of Medicine and Health Sciences, Norwegian University of Science and Technology, Trondheim, Norway; 5 Department of Physical Performance, Norwegian School of Sport Sciences, Oslo, Norway; 6 Department of Sports, Physical Education and Outdoor Studies, Faculty of Humanities, Sports and Educational Science, University of South-Eastern Norway, Vestfold, Norway; 7 Faculty of Health and Welfare, Østfold University College, Fredrikstad, Norway

**Keywords:** Elderly, Independent living, Strength training, Home-based exercise, Functional mobility, Elastic band

## Abstract

**Background:**

Aging is associated with reduced muscle mass and strength leading to impaired physical function. Resistance training programs incorporated into older adults’ real-life settings may have the potential to counteract these changes. We evaluated the effectiveness of 8 months resistance training using easily available, low cost equipment compared to physical activity counselling on physical function, muscle strength, and body composition in community-dwelling older adults receiving home care.

**Methods:**

This open label, two-armed, parallel group, cluster randomized trial recruited older adults above 70 years (median age 86.0 (Interquartile range 80–90) years) receiving home care. Participants were randomized at cluster level to the resistance training group (RTG) or the control group (CG). The RTG trained twice a week while the CG were informed about the national recommendations for physical activity and received a motivational talk every 6th week. Outcomes were assessed at participant level at baseline, after four, and 8 months and included tests of physical function (chair rise, 8 ft-up-and-go, preferred- and maximal gait speed, and stair climb), maximal strength, rate of force development, and body composition.

**Results:**

Twelve clusters were allocated to RTG (7 clusters, 60 participants) or CG (5 clusters, 44 participants). The number of participants analyzed was 56–64 (6–7 clusters) in RTG and 20–42 (5 clusters) in CG. After 8 months, multilevel linear mixed models showed that RTG improved in all tests of physical function and maximal leg strength (9–24%, *p* = 0.01–0.03) compared to CG. No effects were seen for rate of force development or body composition.

**Conclusion:**

This study show that resistance training using easily available, low cost equipment is more effective than physical activity counselling for improving physical function and maximal strength in community-dwelling older adults receiving home care.

**Trial registration:**

ISRCTN1067873

## Background

Aging is associated with reduced muscle mass [1] and strength [1, 2] followed by a decline in physical function (e.g., ability to walk, rise from a chair, walk stairs) [2]. Furthermore, the ability to generate force rapidly decreases more than maximal strength in older adults [2, 3], and it has been argued that power and rate of force development (RFD) is more important for physical function and the ability to carry out activities of daily life [3, 4]. To promote healthy aging and the ability to live independently in aging populations, it is essential to identify effective strategies to counteract or delay these age-related changes.

Resistance training has proven to be safe and effective to counteract loss of muscle mass [5], strength [5, 6], and physical function in older adults [6, 7]. Resistance training is often performed at fitness centers using resistance training machines and free weights [4, 7]. However, approaches that can be easily incorporated into real-life settings have been called upon, as lack of availability, training experience, and affordability may limit older adults’ access to traditional training facilities [8, 9]. One possibility is to provide resistance training programs using easily available, low-cost equipment such as elastic bands, body weight, and other equipment (e.g., ankle weights, water canes) [10, 11]. Such equipment facilitates incorporation of resistance training in real-life settings. However, studies using such training programs show inconclusive results with respect to improvements in maximal strength [12–19] and body composition in older adults [14, 17, 20, 21], as well as their transferability to physical function [12, 13, 15, 16, 18–23]. Furthermore, few studies have investigated the effect of resistance training programs using easily available, low cost equipment on the ability to generate force rapidly and the results have been inconsistent [15, 16].

Most studies have examined healthy, community-dwelling older adults below the age of 80 years or those living in an institution [7, 10–12, 14, 17, 18, 20–22]. The oldest old (> 80 years) still living at home while receiving home care services remains understudied [15, 23], Effective interventions for this population could provide a golden window of opportunity to promote independent living by improving physical function and muscle strength. With an increasing older population, easily available, low-cost training programs might reduce the need for home care services. Thus, this cluster randomized trial examined the effectiveness of an 8 months resistance training program using easily available, low cost equipment, compared to a control group receiving physical activity counselling, on physical function, muscle strength, and body composition in community-dwelling older adults receiving home care. We hypothesized that greater improvements in physical function and muscle strength would be demonstrated in the resistance training group than the control group.

## Methods

### Trial design

The Independent Self-Reliant Active Elderly (ISRAE) study is an open label, two-armed, parallel group cluster randomized controlled trial (RCT), conducted in three municipalities (Sogndal, Leikanger and Luster) in Western Norway, from August 2016 to August 2018. A cluster RCT was chosen to avoid contamination and to increase adherence. Participants were divided into 12 clusters (range 5–16 participants) where participants living in the same geographical area belonged to the same cluster. The clusters were allocated (3:2 ratio) to the resistance training group (RTG) or the control group (CG) receiving physical activity counselling. The intervention lasted for 8 months and participants were followed for 1 year after the end of the intervention. Here, we report the intervention effects at the participant level on physical function tests, maximal strength, RFD, and body composition four and 8 months after study inclusion.

The Regional Committee for Medical and Health Research Ethics and the Norwegian Centre for Research Data approved the study (2016/51 and 49,361/s/AGH, respectively), and it was conducted in accordance with the Declaration of Helsinki. Due to changes in design, the study was registered retrospectively (ISRCTN registry:1067873) and is reported according to the Consort statement extension to cluster RCTs [24]. Oral and written information about the study was given to all participants and written informed consent was signed before randomization.

### Participants

Participants were recruited through the health care services in the three municipalities. Older adults above 70 years, living at home, and receiving home care due to functional and/or medical disabilities were eligible for inclusion. Participants were excluded if they had serious cognitive impairment (e.g. Alzheimer’s disease, dementia), physical diagnoses/conditions that could affect testing or training, and/or disapproval from a medical doctor due to contraindications for training. During inclusion, an amendment was made to the inclusion criteria; seven individuals otherwise meeting the eligibility criteria, but were below 70 years (median 67 (range 63–69) years) were included in the study to increase the sample size.

### Intervention

RTG was offered a resistance training program twice per week for 8 months, from end of September 2016 to the end of May 2017. The intervention was targeted at the participant level. Each session lasted for 30–45 min and was supervised by trained exercise instructors. Training was performed in groups at the local health care centers using easily available, low cost equipment such as elastic bands (ROPES a/s, Aasgardstrand, Norway), body weight, and water canes. The included exercises aimed to strengthen the muscle groups most important for daily living activities (Table 1). To ensure progression, number of series and repetitions were manipulated, and new exercises were introduced (Table 1). Furthermore, the exercise instructors tailored the intensity to the individual by using chairs, adding water canes, and/or changing the thickness and tension (level of pre-stretch) of the elastic band. After the baseline testing, a 5 week introductory phase was conducted, focusing on proper execution of the exercises without going to fatigue. After this, volume and intensity were increased progressively and participants were encouraged to perform each exercise to fatigue – i.e. they were unable to complete more repetitions with proper technique. Participants were encouraged to train with high intentional velocity during the concentric phase (to increase RFD) and with slower controlled velocity in the eccentric phase (to increase the hypertrophic stimulus).. Additionally, participants were encouraged to continue their normal daily activity. Attendance to the resistance training was registered and defined as percentage of sessions met of sessions offered.

**Table 1:** Progression of the resistance training program

Phase	Length (Weeks)	Number of exercises	Description of exercises	Series	Repetitions performed
1	5	5	Rowing, chest press, squats, biceps curl, knee extension	2	10-12^b^
2	10	5	Same as phase 1	3	10–12
3	10	6	Same as phase 1 + shoulder press	3	8–10
4	10	7	Same as phase 3 + up-and-go^a^	4	8–10

^a^ Rising from a chair, walking 3 m and turning around a cone, walking back and sitting down

^b^ Introductory phase, repetitions not performed until fatigue

Participants allocated to CG received counselling on the national recommendations for physical activity and a physical education booklet from the Ministry of Health and Care Services. A researcher or research assistant contacted participants every 6th week by phone or a visit, reminding them about the national recommendations for physical activity and motivating them to stay active.

### Outcomes

Testing was conducted at the health care centers by assessors who were not blinded to allocation.. All outcomes were measured at the participant level.

#### Physical function

Five tests in random order were used to assess physical function. All tests were performed two or three times. Verbal encouragement was given. Time was measured using a stopwatch. Participants could use crutches, walker, and/or armrests of the chair and stairs if necessary. Use of assistive devices was registered at baseline, four, and 8 months to ensure similar test conditions throughout. If the registration of assistive devices was incomplete, the measurement was registered as missing.

##### Chair rise

The test measures the time needed to complete five sit-to-stand cycles [15]. A straight back chair with armrests was used and participants were told to rise to a fully extended position and sit back down five times. The best trial was used for analysis. Coefficient of variation (CV) ranged from 10 to 14%.

##### 8 ft-up-and-go

The test measures the time needed to rise from a chair, walk 2.4 m, turn, walk back to the chair, and sit down [25]. A straight backrest chair with armrests was used. The best trial was used for analysis. CV ranged from 8 to 12%.

##### Gait speed

Preferred and maximal gait speed (m/s) was assessed over 20 m [15]. For preferred speed, participants were instructed to walk in a comfortable pace, while for the maximal speed they were instructed to walk as fast as possible without running. Participants started approximately one meter before and slowed down one meter after the 20 m course. The mean of the trials was used for preferred gait speed while the best trial was used for maximal gait speed. CV ranged from 5 to 8%,

##### Stair climb

The test measures the time needed to walk up a flight of stairs. As testing was conducted at the different health care centers, the same staircase was not used for all participants. However, each participant walked the same staircase at all three test times. The number of steps ranged from 16 to 24, with a vertical climb of 2.7 to 4.0 m. Participants were instructed to ascend the staircase in the same way as they normally would. The best trial was used for analyses. One cluster (CG *n* = 15) did not have access to stairs at their health center and was not included in analyses. CV ranged from 6 to 8%.

#### Maximal strength and rate of force development

Muscle strength was measured during maximal voluntary isometric contraction (MVC) of the knee extensors. Participants were seated in a custom-made flexi-bench (Pivot 430 Flexi-bench, Sportsmaster, Norway) and a non-elastic band (ROPES a/s, Aasgardstrand, Norway) attached to a force cell (Ergotest A/S, Porsgrunn, Norway) was used to measure force development. The knee angle was 90-degrees and the band was placed around the preferred ankle [15]. Two to three trials were performed separated by a 1-min resting period and the best trial was used for analysis. Participants were instructed to contract as “fast and forcefully as possible” for at least 5 s and verbal encouragement was given. Maximal strength (MVC) was defined as the highest mean force output over a 3-s window. RFD was calculated over a 200-millisecond window at the steepest vertical force generation [15]. CV for maximal strength and RFD was 8–9 and 28%, respectively.

#### Grip strength

Grip strength (CV = 5–6%) was measured using a hand-held dynamometer (Baseline® Hydraulic Hand Dynamometer, Elmsford, NY, USA). The participants were instructed to squeeze as hard as they could for three to 5 s using the preferred arm. Verbal encouragement was given. The best of three trials was used for analyses.

#### Body composition

Height was measured without shoes using a stadiometer. Body composition (body mass index (BMI), percentage body fat, and fat free mass) was measured barefooted and in light clothes with bioelectrical impedance analysis using a Tanita weight (Tanita MC 780MA S, Illinois, USA). Participants with a pacemaker did not perform the bioelectrical impedance analysis.

### Randomization and blinding

Randomization was done at cluster level in a 3:2 ratio and carried out in Excel by the project leader using the following procedure: (i) each cluster was given a number [1–16] and large clusters (> 10 participants) were weighted with two numbers. (ii) A random numbers table was used to allocate clusters to RTG based on the assigned numbers (iii). The procedure was stopped when RTG consisted of 60% of the participants (i.e. seven clusters). (iv) The remaining five clusters (40% of participants) were allocated to CG.

For practical reasons none of the researchers or research assistants were blinded. Further, due to the nature of the intervention it was not possible to blind participants or exercise instructors.

### Statistical analysis

Analyses were performed according to the intention to treat principle. The between-group effects were analyzed using multilevel linear mixed models. The baseline level was obtained by merging the two groups [26] and we included the interaction between group and time (baseline, four, and 8 months;2 groups × 3 times). Cluster and participant-id were entered as random effects, accounting for cluster randomization and dependency of repeated measures. Visual inspection of the residuals of outcomes was used to assess normality. Non-normal outcomes were transformed using the natural logarithmic scale. These outcomes were back transformed using the formula exp.(μ + $\frac{\sigma^2}{2}$) to obtain the arithmetic mean estimates. The estimates from the analyses were used to predict outcomes for the two groups at the different time points.

A per-protocol analysis including participants with ≥60% attendance to exercise sessions was conducted. In addition, sensitivity analyses were performed. First, an analysis was conducted including participants who met the original inclusion criteria for age (i.e. ≥ 70 years). Second, for the physical function outcomes, we performed an analysis with adjustment for the baseline value of the outcome without using combined baseline. Lastly, we adjusted stair climb for i) vertical climb and ii) number of steps.

For normally distributed variables, descriptive data is presented as mean and standard deviation (SD) and results are presented as mean and 95% confidence interval (95% CI). For non-normal variables, descriptive data is presented as median and 25–75 percentile (Interquartile range, IQR) unless stated otherwise. The estimated mean difference between two groups represents the ratio of the geometric mean for RTG to the geometric mean for CG [27] and are presented as ratios and 95% CI. Intra-cluster correlation coefficients (ICC) were calculated for all outcomes as between cluster variation divided by total variation [28]. Cohen’s d effect sizes and 95% CIs were calculated for between-group changes from baseline to four and 8 months. An effect size of 0.2, 0.5, and 0.8 was considered small, medium, and large, respectively [29]. A *p*-value of < 0.05 was considered statistically significant. All analyses were performed in STATA 15 (StataCorp. 2017. Stata Statistical Software: Release 15. College Station, TX: StataCorp LLC).

## Results

**Fig. 1:**
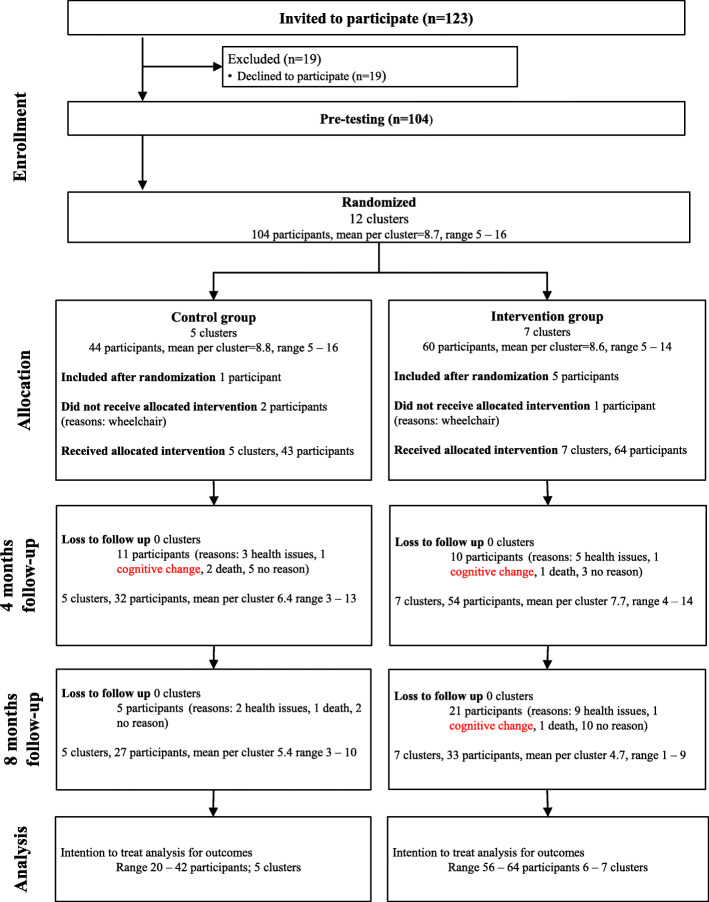
Flow of participants through the study

We invited 123 older adults fitting the inclusion criteria to participate in the study, 104 met for baseline testing and were divided into 12 clusters eligible for randomization. Six participants were included after randomization and assigned to a cluster based on their geographical residency. Three participants using wheelchairs could not perform testing and were excluded. Number of participants and clusters analyzed was 76–106 and 11–12, respectively. The flow of participants through the study is illustrated in Fig. 1. There were no adverse events reported in any of the groups.

### Participant characteristics

Baseline characteristics of the groups are presented in Table 2. The median age was 86 (80–90) years and the majority were women (60%). Most of the participants used assistive walking devices (60%). The mean attendance to training sessions was 51%. The dropout rate was 44% (RTG *n* = 31; CG *n* = 16) and those dropping out were somewhat older (median age 88 (83–91) years), with similar representation of males and females (55% females).

**Table 2:** Baseline characteristics of participants

Characteristics	RTG (*n* = 64)	CG (*n* = 43)	ICC
Age (years) median (IQR)	86.5 (80–90)	86.0 (80–90)	
Sex			
Female n (%)	42 (66)	22 (51)	
Use of walking aids n (%)*	33 (52)	31 (72)	
Height (cm) mean (SD)	160 (9)	164 (9)	
Body mass (kg) median (IQR)	66.5 (55.5–79.5)^a^	70.4 (62.4–80.2)^b^	
Body Mass Index (kg/m^2^) median (IQR)	25.1 (23.6–28.1)^a^	27.0 (23.7–30.3)^b^	0.00
Fat mass (%) median (IQR)	29.5 (24.4–37.4)^c^	30.4 (23.4–38.2)^d^	0.05
Fat free mass (kg) median (IQR)	42.9 (37.5–55.3)^e^	51.3 (43.2–61.2)^f^	0.02
Chair rise (s) median (IQR)	16.0 (12.7–20.7)^a^	19.3 (16.9–24.3)^g^	0.01
8 ft. up and go (s) median (IQR)	11.9 (8.5–18.6)^h^	16.0 (10.7–19.7)^i^	0.07
Stair walk (s) median (IQR)	18.8 (12.7–29.3)^j^	23.1 (19.0–33.6)^k^	0.00
Preferred gait speed (m/s) mean (SD)	0.78 (0.28)^a^	0.66 (0.18)^l^	0.07
Maximal gait speed (m/s) mean (SD)	1.1 (0.43)^a^	0.9 (0.28)^l^	0.06
Leg MVC (N) mean (SD)	185 (82)^a^	175 (67)^g^	0.00
Leg MVC relative (N/kg) mean (SD)	2.8 (1.0)^h^	2.3 (0.8)^m^	0.00
Leg RFD (N/s) mean (SD)	406 (323)^a^	447 (279)^g^	0.10
Grip strength (kg) mean (SD)	25.4 (8.1)	28.0 (7.8)^g^	0.00

*RTG* Resistance training group, *CG* Control group, *ICC* Intra cluster correlation, *MVC* Maximal voluntary isometric contraction, *RFD* Rate of force development, *IQR* Interquartile range 25- to 75 percentile, *SD* Standard deviation, *N* Newton

*Includes walker or crutches. One participant in CG with missing data

^a^*n* = 63 ^b^*n* = 40 ^c^*n* = 59 ^d^*n* = 36 ^e^*n* = 58 ^f^*n* = 31 ^g^*n* = 42 ^h^*n* = 62 ^i^*n* = 41 ^j^*n* = 55 ^k^*n* = 20 ^l^*n* = 41 ^m^*n* = 39

### Physical function

**Fig. 2:**
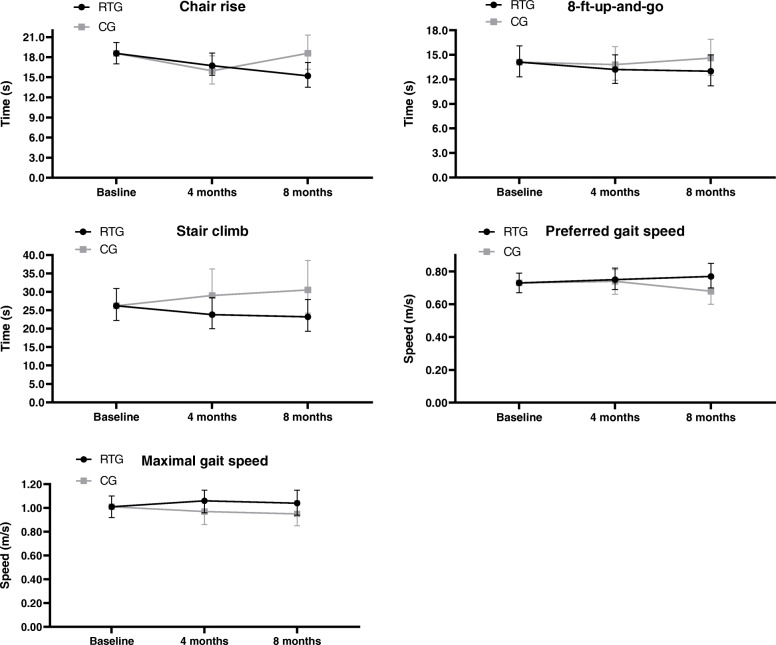
Changes in physical function from baseline through four and eight months. Values are estimated means and 95% confidence intervals

Figure 2 show changes in physical function from baseline to four and 8 months. After 4 months, RTG improved in stair climb (18%, *p* = 0.03) and maximal gait speed (8%, *p* = 0.01) compared to CG (Table 3, Fig. 2). No other statistically significant between-group differences were found after 4 months. After 8 months, RTG improved in all physical function tests (9–24%, p = 0.01–0.03) compared to CG (Table 3, Fig. 2). Supplementary Table S1 shows between-group effect sizes.

**Table 3:** Physical function, strength and body composition from baseline to four and eight months

Outcome	Analyzed		Baseline Mean (95% CI)	4 months		Between-group difference		8 months		Between-group difference	
	RTG n	CG n		RTG Mean (95% CI)	CG Mean (95% CI)	Mean (95% CI)	*p*	RTG Mean (95% CI)	CG Mean (95% CI)	Mean (95% CI)	*p*
Chair rise (s)^a^	63	42	18.6 (17.0–20.2)	16.7 (15.1–18.6)	16.0 (14.0–18.2)	1.05 (0.91–1.20)	0.500	15.2 (13.5–17.2)	18.6 (16.2–21.3)	0.81 (0.70–0.96)	0.010
8 ft-up-and-go (s)^a^	63	41	14.1 (12.3–16.1)	13.2 (11.5–15.0)	13.8 (11.9–16.0)	0.96 (0.87–1.05)	0.350	13.0 (11.2–15.0)	14.6 (12.5–16.9)	0.89 (0.80–0.99)	0.030
Stair climb (s)^a^	56	20	26.2 (22.2–30.9)	23.8 (20.0–28.4)	29.0 (23.3–36.2)	0.82 (0.69–0.98)	0.030	23.2 (19.3–27.9)	30.5 (24.2–38.5)	0.76 (0.62–0.93)	0.007
Preferred gait speed (m/s)^a^	63	41	0.73 (0.67–0.79)	0.75 (0.69–0.82)	0.74 (0.66–0.81)	0.01 (−0.04–0.07)	0.600	0.77 (0.70–0.85)	0.68 (0.60–0.76)	0.09 (0.03–0.16)	0.006
Maximal gait speed (m/s)^a^	63	41	1.01 (0.92–1.10)	1.06 (0.96–1.15)	0.97 (0.86–1.07)	0.09 (0.02–0.16)	0.010	1.04 (0.94–1.15)	0.95 (0.85–1.06)	0.09 (0.00–0.17)	0.030
Grip strength (kg)	64	42	26.4 (24.7–28.0)	26.5 (24.6–28.4)	24.9 (22.7–27.0)	1.2 (−0.5–3.7)	0.134	22.7 (20.5–24.9)	23.3 (21.0–25.5)	−0.6 (−3.0–1.9)	0.639
Leg MVC (N)	64	42	181 (166–195)	195 (179–212)	179 (160–198)	16 (−2–34)	0.074	201 (182–219)	175 (155–194)	26 (6–46)	0.010
Leg MVC relative (N/kg)	63	42	2.6 (2.4–2.8)	2.8 (2.6–3.0)	2.6 (2.3–2.8)	0.2 (−0.02–0.5)	0.073	2.9 (2.7–3.1)	2.5 (2.3–2.8)	0.4 (0.1–0.7)	0.005
Leg RFD (N/s)	64	42	431 (365–497)	436 (356–517)	337 (241–434)	99 (−8–205)	0.069	384 (292–476)	383 (282–483)	1 (− 118–120)	0.982
BMI (kg/m^2^)^a^	63	43	26.4 (25.4–27.5)	26.6 (25.5–27.7)	26.5 (25.4–27.7)	1.00 (0.98–1.02)	0.890	26.3 (25.2–27.4)	26.5 (25.3–27.6)	0.99 (0.97–1.02)	0.600
Fat mass (%)^a^	59	37	28.9 (26.6–31.4)	28.9 (26.4–31.7)	27.8 (25.0–30.9)	1.04 (0.95–1.14)	0.380	28.2 (25.5–31.2)	29.7 (26.7–33.0)	0.95 (0.86–1.05)	0.310
Fat free mass (kg)^a^	59	35	47.2 (44.6–49.9)	47.5 (44.9–50.3)	47.1 (44.4–49.9)	1.01 (0.99–1.03)	0.390	46.8 (44.2–49.6)	47.1 (44.4–49.9)	0.99 (0.97–1.02)	0.670

Estimated means and 95% confidence intervals (95% CI) using linear mixed models (unadjusted model). ^a^ Between-group differences for transformed variables are presented as ratio of the geometric mean for RTG to the geometric mean for CG with corresponding 95% CI

*RTG* Resistance training group, *CG* Control group, *MVC* Maximal voluntary contraction, *RFD* Rate of force development, *N* Newton

### Maximal strength and rate of force development

There were no statistically significant between-group differences after 4 months (Table 3). After 8 months, RTG improved in leg- (18%, *p* = 0.03) and relative leg (16%, *p* = 0.01) MVC strength compared to CG (Table 3). No statistically significant between-group differences were found after 8 months for leg RFD and grip strength. Supplementary Table S1 shows between-group effect sizes.

### Body composition

No statistically significant between-group differences were found after four or 8 months for body composition (Table 3). Supplementary Table S1 shows between-group effect sizes.

### Per protocol- and sensitivity analyses

Following the per-protocol analysis, the between-group difference in 8 ft-up-and-go after 8 months was slightly smaller and no longer statistically significant (10%, *p* = 0.06). The per-protocol analyses did not change the other findings (Supplementary Table S2). After removing participants under 70 years, the between-group difference was slightly smaller and no longer statistically significant for stair climb (17%, *p* = 0.06) after 4 months, and maximal gait speed (7%, *p* = 0.11) and leg MVC (13%, *p* = 0.05) after 8 months (Supplementary Table S3). No other changes were demonstrated following the sensitivity analyses (Supplementary Table S4-S5).

## Discussion

Among community-dwelling older adults receiving home care, resistance training using easily available, low cost equipment improved physical function (chair-rise, 8 ft-up-and-go, stair climb, preferred- and maximal gait speed) and maximal leg strength after 8 months compared to physical activity counselling. Smaller and fewer between-group differences were observed at 4 months. We found no between-group differences for explosive leg strength, grip strength, or body composition.

Our findings are in line with a systematic review and a meta-analysis reporting small to moderate improvements in physical function and muscle strength in older adults following comparable resistance training programs [10, 11]. In a previous study on institutionalized older adults (mean age 83 years), no effects were found for maximal leg strength, however, the number of chair stand repetitions increased in the resistance training group compared to a control group after 6 months of training [13]. Another study including older adults in their 80s and 90s receiving home care found no effects on physical function nor maximal strength after 10 weeks of resistance training using elastic bands, body weight, and water canes [15]. This finding is supported by a study using a comparable sample [23]. The lack of effect on RFD and body composition we observed is consistent with other studies using comparable resistance training programs in older adults (e.g., elastic bands, body weight) [14, 15]. However, some studies including older adults in their 60s and 70s have reported reduced body fat and improved muscle mass [20, 21].

The lack of consistent findings could be explained by several study-differences, such as differences in protocols and outcomes for physical function and muscle strength, training volume and duration, and populations that are not entirely comparable (e.g., different health statuses). These issues are discussed more specifically below.

Our participants had little or no previous experience with resistance training. The design of the training program was in line with recommendations for resistance training programs for older adults [30]. However, the training volume (2 × 30-45 min/week) and attendance to training sessions (51%) might have been too low to produce enough long-term training stimuli, especially for muscle growth [31]. Low volume and intensity, especially the first 5 weeks, could further explain the fewer and smaller effects seen after four compared to 8 months of training. The larger effects seen after 8 months could indicate that training duration is of importance for this group of older adults (> 80 years). Furthermore, elastic bands provide light resistance that increases at the end of the range of motion [32] and greater force is generated during the last half of the concentric phase, when the velocity is lower. Thus, characteristics of the elastic bands may limit the ability to effectively load the muscles in the concentric phase with high velocity. The lack of training effect for RFD could also be explained by large within subject variation in RFD. Training specificity could also explain our findings, as several of the included exercises (e.g., the squat, knee-extension, and up-and-go) are highly transferrable to the physical function- and strength tests. Lastly, CG was more disabled (worse performance on physical function tests and more use of walking aids) and more overweight compared to RTG at baseline. This could hamper the ability to find between-group differences due to regression towards the mean [33]. Furthermore, the higher fat free mass in CG at baseline could explain the lack of training effect on muscle growth.

An effective resistance training program could reduce the need for home care, thereby promoting independent living. This resistance training program used easily available, low-cost equipment, making it feasible and possible to implement in real-life settings of older adults (e.g., health care centre or at home). Importantly, the decreased performance seen in CG from baseline to 8 months, but not in RTG, could indicate that reductions in physical function and muscle strength can be counteracted in the oldest old (> 80 years). However, after training, RTG still demonstrated a preferred gait speed below what has been recommended to represent good health and physical function in older adults (≥1.0 m/s) [34]. Furthermore, RTG did not reach normative age-thresholds (80–90 years) for 8 ft-up-and-go (5.2–9.6 s) [25]. Thus, whether the improvements demonstrated are transferrable to improving independence are unknown. We therefore speculate that action should be made before older adults are at the threshold for institutionalization. Whether greater benefits are achievable in a comparable sample by increasing volume and intensity should be investigated. Furthermore, we experienced a large dropout (44%) and low attendance, which was not surprising given the age and health status of the participants, as shown by others [35, 36]. Future studies should include strategies aimed at maximizing compliance, such as strengthening older adults’ self-efficacy and motivation [36]. Future research should also evaluate the effect of earlier implementation, as well as the cost-effectiveness of implementing long-term resistance training in older adults’ real-life settings.

The main strength of the study is its ecological validity with the long-term resistance training program utilizing easily availed, low cost equipment that could be incorporated into older adults’ real-life settings. Additionally, the inclusion of participants receiving home care, possibly representing a window of opportunity for delaying institutionalization strengthens our study. Some limitations need to be addressed. First, the participants varied in age, physical function, and use of assistive walking devices, thus, the generalizability of the findings are unknown. Second, six participants were included after randomization, possibly biasing group allocation. Third, nutritional intake, quality of nutrition, and hydration was not standardized before testing, reducing the sensitivity to detect subtle changes in body composition. Fourth, dropout was high, but we used multilevel mixed models, which have the strength of handling missing data without imputation [26]. However, these models rely on the assumption of “missing at random” and we can not disregard the possibility of bias due to loss to follow up. Fifth, the sample is small for a cluster RCT and future studies with higher statistical power should be carried out. Lastly, some caution should be made when interpreting our findings due to multiple testing and lack of blinding.

## Conclusion

In community-dwelling older adults receiving home care, resistance training improved all measures of physical function and maximal leg strength after 8 months compared to physical activity counselling. No effects were found for RFD, grip strength, or body composition. These findings suggest that resistance training programs utilizing easily available, low cost equipment could be beneficial to implement in real-life settings of community-dwelling older adults.

## Supplementary information

**Additional file 1:.** Between-group Cohens’ d effect sizes and 95% confidence intervals. This additional file is a one page table (.docx) showing the Cohens’ d effect sizes for between-group differences for all outcomes.

**Additional file 2:.** Per protocol analysis including participants with ≥60% attendance to training sessions. Values are estimated means and 95% confidence intervals (95% CI), unless stated otherwise. This additional file is a table (.docx) showing results from the per protocol analysis (participants with ≥60% attendance to training) for all outcomes.

**Additional file 3:.** Sensitivity analysis including only participants over the age of 70 years. Values are estimated means and 95% confidence intervals (95% CI), unless stated otherwise. This additional file is a table (.docx) showing results from the sensitivity analysis including only the participants ≥70 years, as first intended by the inclusion criteria.

**Additional file 4:.** Sensitivity analysis of physical function outcomes without combined baseline, but adjusted for baseline differences of the outcome. Values are estimated means and 95% confidence intervals (95% CI), unless stated otherwise. This additional file is a table (.docx) showing results from the sensitivity analysis without using combined baseline, but adjusting for the baseline differences of the outcome. This sensitivity analysis was performed only for outcomes of physical function.

**Additional file 5:.** Sensitivity analysis of stair climb, adjusted for vertical climb and number of steps. Values are estimated means and 95% confidence intervals (95% CI), unless stated otherwise. This additional file is a table (.docx) showing results from the sensitivity analysis of stair climb, where we adjusted for vertical climb in one model and number of steps in another model.

## Data Availability

The dataset used and analyzed during the current study is available from the corresponding author upon reasonable request.

## Abbreviations

RT: The resistance training group
CG: The control group
MVC: Maximal voluntary isometric contraction
RFD: Rate of force development
N: Newton
N/kg: Newton per kilogram
m/s: Meters per second
SD: Standard deviation
IQR: Interquartile range
ICC: Intra-cluster correlation coefficient
CI: Confidence interval
CV: Coefficient of variation
