# Correction to: Effectiveness of a resistance training program on physical function, muscle strength, and body composition in community-dwelling older adults receiving home care: a cluster-randomized controlled trial

**DOI:** 10.1186/s11556-020-00245-7

**Published:** 2020-09-10

**Authors:** Hilde Bremseth Bårdstu, Vidar Andersen, Marius Steiro Fimland, Lene Aasdahl, Truls Raastad, Kristoffer T. Cumming, Atle Hole Sæterbakken

**Affiliations:** 1 Department of Sport, Food and Natural Sciences, Faculty of Education, Arts and Sports, Western Norway University of Applied Sciences, PB 133, 6851 Sogndal, Norway; 2 Department of Neuromedicine and Movement Science, Faculty of Medicine and Health Sciences, Norwegian University of Science and Technology, Trondheim, Norway; 3 Unicare Helsefort Rehabilitation Centre, Rissa, Norway; 4 Department of Public Health and Nursing, Faculty of Medicine and Health Sciences, Norwegian University of Science and Technology, Trondheim, Norway; 5 Department of Physical Performance, Norwegian School of Sport Sciences, Oslo, Norway; 6 Department of Sports, Physical Education and Outdoor Studies, Faculty of Humanities, Sports and Educational Science, University of South-Eastern Norway, Vestfold, Norway; 7 Faculty of Health and Welfare, Østfold University College, Fredrikstad, Norway

**Correction to: Eur Rev Aging Phys Act 17, 11 (2020)**

**https://doi.org/10.1186/s11556-020-00243-9**

Following publication of the original article [1], the authors noticed that the red text in Fig. 1 was not removed during proofing stage. The original article [1] has been updated.

The correct Fig. 1 is shown below.

**Fig. 1:**
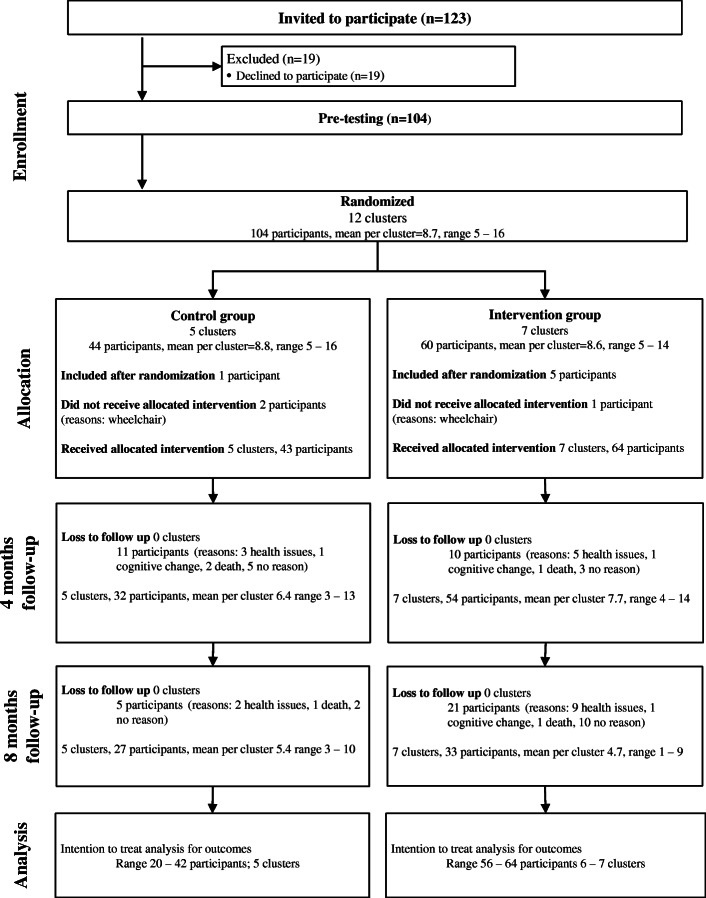
Flow of participants through the study
