# Supplementary material for: Effectiveness of a resistance training program on physical function, muscle strength, and body composition in community-dwelling older adults receiving home care: a cluster-randomized controlled trial
Source: Eur Rev Aging Phys Act. 2020 Aug 7;17:11. doi: 10.1186/s11556-020-00243-9 (PMC7414534; doi:10.1186/s11556-020-00243-9)
Supplement: Supplementary file 1 — Additional file 1:. Between-group Cohens’ d effect sizes and 95% confidence intervals. This additional file is a one page table (.docx) showing the Cohens’ d effect sizes for between-group differences for all outcomes. [file 11556_2020_243_MOESM1_ESM.docx]

Table S1 Between group Cohens’ d effect sizes and 95% confidence intervals (95% CI).

| Outcome | RTG compared with CG | |
| --- | --- | --- |
|  | 4 months | 8 months |
| Chair rise (s) | -0.12 (-0.24- 0.48) | -0.52 (-0.93- -0.11) |
| 8ft-up-and-go (s) | -0.09 (-0.29- -0.10) | -0.24 (-0.45- -0.03) |
| Stair climb (s) | -0.29 (-0.56- -0.02) | -0.41 (-0.70- -0.11) |
| Preferred gait speed (m/s) | 0.06 (-0.17- 0.28) | 0.36 (0.10- 0.62) |
| Maximal gait speed (m/s) | 0.23 (0.05- 0.41) | 0.24 (0.02- 0.45) |
| Grip strength (kg) | 0.20 (-0.06- 0.47) | -0.07 (-0.38- 0.24) |
| Leg MVC (N) | 0.21 (-0.02- 0.44) | 0.33 (0.08- 0.59) |
| Leg MVC relative (N/kg) | 0.23 (-0.02- 0.49) | 0.40 (0.12- 0.68) |
| Leg RFD (N/s) | 0.36 (-0.03- 0.74) | 0.01 (-0.43- 0.43) |
| BMI (kg/m^2^) | -0.01 (-0.10- 0.12) | -0.03 (-0.15- 0.09) |
| Fat mass (%) | 0.11 (-0.14- 0.35) | -0.14 (-0.41- 0.13) |
| Fat free mass (kg) | 0.04 (-0.05- 0.13) | -0.02 (-0.12- 0.08) |

RTG, Resistance training group; CG, Control group; MVC, Maximal voluntary isometric contraction; RFD, Rate of force development; N, Newton.
