# Supplementary material for: Effectiveness of a resistance training program on physical function, muscle strength, and body composition in community-dwelling older adults receiving home care: a cluster-randomized controlled trial
Source: Eur Rev Aging Phys Act. 2020 Aug 7;17:11. doi: 10.1186/s11556-020-00243-9 (PMC7414534; doi:10.1186/s11556-020-00243-9)
Supplement: Supplementary file 2 — Additional file 2:. Per protocol analysis including participants with ≥60% attendance to training sessions. Values are estimated means and 95% confidence intervals (95% CI), unless stated otherwise. This additional file is a table (.docx) showing results from the per protocol analysis (participants with ≥60% attendance to training) for all outcomes. [file 11556_2020_243_MOESM2_ESM.docx]

Table S2 Per protocol analysis including participants with ≥60% attendance to training sessions.

| Outcome | Analyzed | | Baseline  Mean  (95% CI) |  | 4 months | |  | Between-group  difference | |  | 8 months | |  | Between-group difference | |
| --- | --- | --- | --- | --- | --- | --- | --- | --- | --- | --- | --- | --- | --- | --- | --- |
|  | RTG  n | CG  n |  |  | RTG  Mean  (95% CI) | CG  Mean  (95% CI) |  | Mean  (95% CI) | *p* |  | RTG  Mean  (95% CI) | CG  Mean  (95% CI) |  | Mean  (95% CI) | *p* |
| Chair rise (s)^a^ | 27 | 42 | 18.4  (16.6-20.4) |  | 15.8  (13.7-18.1) | 16.4  (14.2-18.9) |  | 0.96  (0.81-1.15) | 0.670 |  | 14.7  (12.7-17.1) | 19.7  (17.0-22.9) |  | 0.75  (0.62-0.90) | 0.003 |
| 8ft-up-and-go (s)^a^ | 24 | 41 | 13.5  (11.6-15.7) |  | 12.8  (10.9-15.0) | 13.3  (11.3-15.6) |  | 0.96  (0.87-1.06) | 0.468 |  | 12.6  (10.8-14.8) | 14.0  (11.9-16.5) |  | 0.90  (0.81-1.00) | 0.056 |
| Stair climb (s)^a^ | 26 | 20 | 22.6  (17.8-28.7) |  | 21.1  (16.4-27.2) | 27.7  (20.8-36.7) |  | 0.76  (0.61-0.94) | 0.015 |  | 20.4  (15.8-26.5) | 30.8  (23.0-41.3) |  | 0.66  (0.52-0.84) | 0.001 |
| Preferred gait speed (m/s) | 27 | 41 | 0.73  (0.66-0.79) |  | 0.78  (0.71-0.85) | 0.74  (0.67-0.81) |  | 0.04  (-0.01-0.10) | 0.144 |  | 0.78  (0.71-0.86) | 0.68  (0.61-0.75) |  | 0.10  (0.04-0.16) | 0.001 |
| Maximal gait speed (m/s) | 27 | 41 | 1.02  (0.91-1.13) |  | 1.07  (0.95-1.19) | 0.98  (0.86-1.09) |  | 0.09  (0.02-0.17) | 0.010 |  | 1.04  (0.92-1.16) | 0.96  (0.84-1.08) |  | 0.08  (0.00-0.16) | 0.047 |
| Grip strength (kg) | 27 | 42 | 27.5  (25.1-29.9) |  | 27.1  (24.3-29.9) | 25.8  (23.0-28.6) |  | 1.3  (-1.3-3.9) | 0.321 |  | 22.6  (19.6-25.6) | 24.2  (21.4-27.0) |  | -1.6  (-4.5-1.4) | 0.290 |
| Leg MVC (N) | 27 | 42 | 176  (157-195) |  | 191  (169-214) | 176  (154-197) |  | 16  (-5-26) | 0.138 |  | 200  (177-223) | 171  (148-193) |  | 29  (7-51) | 0.010 |
| Leg MVC relative  (N/kg) | 27 | 42 | 2.5  (2.3-2.7) |  | 2.8  (2.5-3.1) | 2.5  (2.2-2.8) |  | 0.3  (-0.0-0.6) | 0.068 |  | 2.9  (2.6-3.2) | 2.4  (2.1-2.7) |  | 0.5  (0.2-0.8) | 0.002 |
| Leg RFD (N/s) | 27 | 42 | 443  (362-523) |  | 448  (343-553) | 346  (243-449) |  | 102  (-20-225) | 0.101 |  | 403  (294-512) | 390  (283-497) |  | 13  (-116-143) | 0.839 |
| BMI (kg/m^2^)^a^ | 27 | 43 | 26.5  (24.9-28.2) |  | 26.8  (25.1-28.6) | 26.6  (24.9-28.4) |  | 1.01  (0.98-1.04) | 0.623 |  | 26.3  (24.6-28.0) | 26.5  (24.9-28.3) |  | 0.99  (0.96-1.02) | 0.497 |
| Fat mass (%)^a^ | 25 | 37 | 28.0  (24.9-31.6) |  | 28.3  (24.7-32.5) | 27.1  (23.6-31.1) |  | 1.05  (0.93-1.18) | 0.458 |  | 27.4  (23.8-31.6) | 28.9  (25.2-33.2) |  | 0.95  (0.84-1.07) | 0.409 |
| Fat free mass (kg)^a^ | 25 | 35 | 47.3  (43.7-51.2) |  | 47.9  (44.2-51.9) | 47.2  (43.5-51.1) |  | 1.02  (0.99-1.04) | 0.241 |  | 46.7  (43.1-50.6) | 47.1  (43.5-51.1) |  | 0.99  (0.96-1.02) | 0.454 |

Estimated means and 95% confidence intervals (95% CI) using linear mixed models (unadjusted model). ^a^ Between-group differences for transformed variables are presented as ratio of the geometric mean for RTG to the geometric mean for CG and 95% CI.

RTG, Resistance training group; CG, Control group; MVC, Maximal voluntary isometric contraction; RFD, Rate of force development; N, Newton.
