# Supplementary material for: Effectiveness of a resistance training program on physical function, muscle strength, and body composition in community-dwelling older adults receiving home care: a cluster-randomized controlled trial
Source: Eur Rev Aging Phys Act. 2020 Aug 7;17:11. doi: 10.1186/s11556-020-00243-9 (PMC7414534; doi:10.1186/s11556-020-00243-9)
Supplement: Supplementary file 3 — Additional file 3:. Sensitivity analysis including only participants over the age of 70 years. Values are estimated means and 95% confidence intervals (95% CI), unless stated otherwise. This additional file is a table (.docx) showing results from the sensitivity analysis including only the participants ≥70 years, as first intended by the inclusion criteria. [file 11556_2020_243_MOESM3_ESM.docx]

Table S3 Sensitivity analysis including participants ≥70 years.

| Outcome | Analyzed | | Baseline  Mean  (95% CI) |  | 4 months | |  | Between-group  difference | |  | 8 months | |  | Between-group difference | |
| --- | --- | --- | --- | --- | --- | --- | --- | --- | --- | --- | --- | --- | --- | --- | --- |
|  | RTG  n | CG  n |  |  | RTG  Mean  (95% CI) | CG  Mean  (95% CI) |  | Mean  (95% CI) | *p* |  | RTG  Mean  (95% CI) | CG  Mean  (95% CI) |  | Mean  (95% CI) | *p* |
| Chair rise (s)^a^ | 58 | 40 | 18.9  (17.4-20.4) |  | 16.9  (15.3-18.8) | 16.3  (14.3-18.5) |  | 1.04  (0.90-1.20) | 0.593 |  | 15.2  (13.4-17.3) | 19.1  (16.7-22.0) |  | 0.80  (0.67-0.94) | 0.008 |
| 8ft-up-and-go (s)^a^ | 58 | 39 | 14.4  (12.6-16.3) |  | 13.3  (11.6-15.3) | 14.0  (12.1-16.2) |  | 0.95  (0.86-1.04) | 0.260 |  | 13.3  (11.5-15.3) | 14.8  (12.8-17.2) |  | 0.90  (0.81-0.99) | 0.038 |
| Stair climb (s)^a^ | 51 | 18 | 27.0  (23.0-31.7) |  | 24.7  (20.8-29.3) | 29.8  (23.7-37.5) |  | 0.83  (0.68-1.01) | 0.063 |  | 24.4  (20.3-29.4) | 31.1  (24.4-39.7) |  | 0.78  (0.63-0.98) | 0.035 |
| Preferred gait speed (m/s) | 58 | 39 | 0.72  (0.66-0.78) |  | 0.74  (0.68-0.81) | 0.73  (0.66-0.80) |  | 0.01  (-0.05-0.08) | 0.658 |  | 0.77  (0.70-0.83) | 0.67  (0.60-0.75) |  | 0.09  (0.02-0.16) | 0.011 |
| Maximal gait speed (m/s) | 58 | 39 | 0.99  (0.90-1.08) |  | 1.04  (0.94-1.13) | 0.95  (0.85-1.05) |  | 0.09  (0.01-0.16) | 0.019 |  | 1.01  (0.91-1.11) | 0.94  (0.83-1.04) |  | 0.07  (-0.02-0.16) | 0.108 |
| Grip strength (kg) | 59 | 40 | 25.3  (24.4-27.5) |  | 25.8  (24.0-27.6) | 24.4  (22.3-26.5) |  | 1.4  (-0.8-3.6) | 0.225 |  | 21.6  (19.3-23.8) | 23.0  (20.8-25.2) |  | -1.4  (-4.1-1.2) | 0.295 |
| Leg MVC (N) | 59 | 40 | 177  (163-191) |  | 190  (174-206) | 176  (157-194) |  | 14  (-4-33) | 0.130 |  | 192  (173-210) | 170  (151-190) |  | 22  (1-43) | 0.050 |
| Leg MVC relative (N/kg) | 58 | 40 | 2.5  (2.3-2.7) |  | 2.8  (2.5-3.0) | 2.6  (2.3-2.8) |  | 0.2  (-0.1-0.4) | 0.128 |  | 2.8  (2.5-3.0) | 2.5  (2.2-2.7) |  | 0.3  (0.02-0.6) | 0.033 |
| Leg RFD (N/s) | 59 | 40 | 405  (343-467) |  | 424  (346-501) | 333  (241-425) |  | 91  (-12-194) | 0.085 |  | 371  (279-462) | 365  (268-461) |  | 6  (-112-124) | 0.924 |
| BMI (kg/m^2^)^a^ | 58 | 41 | 26.4  (25.3-27.5) |  | 26.5  (25.4-27.7) | 26.3  (25.2-27.5) |  | 1.01  (0.99-1.03) | 0.489 |  | 26.2  (25.1-27.4) | 26.4  (25.3-27.6) |  | 0.99  (0.97-1.02) | 0.555 |
| Fat mass (%)^a^ | 54 | 35 | 29.0  (26.5-31.6) |  | 29.2  (26.5-32.2) | 22.0  (24.7-30.8) |  | 1.06  (0.96-1.17) | 0.235 |  | 27.9  (25.0-31.1) | 29.7  (26.6-33.2) |  | 0.94  (0.84-1.05) | 0.255 |
| Fat free mass (kg)^a^ | 54 | 33 | 46.9  (44.4-49.6) |  | 47.1  (44.5-49.8) | 46.6  (44.0-49.3) |  | 1.01  (0.99-1.03) | 0.325 |  | 46.5  (43.9-49.2) | 46.8  (44.2-49.6) |  | 0.99  (0.97-1.02) | 0.616 |

Estimated means and 95% confidence intervals (95% CI) using linear mixed models (unadjusted model). ^a^ Between-group differences for transformed variables are presented as ratio of the geometric mean for RTG to the geometric mean for CG and 95% CI.

RTG, Resistance training group; CG, Control group; MVC, Maximal voluntary isometric contraction; RFD, Rate of force development; N, Newton.
