# Supplementary material for: Effectiveness of a resistance training program on physical function, muscle strength, and body composition in community-dwelling older adults receiving home care: a cluster-randomized controlled trial
Source: Eur Rev Aging Phys Act. 2020 Aug 7;17:11. doi: 10.1186/s11556-020-00243-9 (PMC7414534; doi:10.1186/s11556-020-00243-9)
Supplement: Supplementary file 4 — Additional file 4:. Sensitivity analysis of physical function outcomes without combined baseline, but adjusted for baseline differences of the outcome. Values are estimated means and 95% confidence intervals (95% CI), unless stated otherwise. This additional file is a table (.docx) showing results from the sensitivity analysis without using combined baseline, but adjusting for the baseline differences of the outcome. This sensitivity analysis was performed only for outcomes of physical function. [file 11556_2020_243_MOESM4_ESM.docx]

Table S4 Sensitivity analysis without combined baseline, but adjusted for baseline value of the outcome.

| Outcome | Analyzed | | Baseline | | 4 months | | Between-group  difference | | 8 months | | Between-group difference | | p-value | | |
| --- | --- | --- | --- | --- | --- | --- | --- | --- | --- | --- | --- | --- | --- | --- | --- |
|  | RTG  n | CG  n | RTG  Mean  (95% CI) | CG  Mean  (95% CI) | RTG  Mean  (95% CI) | CG  Mean  (95% CI) | Mean  (95% CI) | *p* | RTG  Mean  (95% CI) | CG  Mean  (95% CI) | Mean  (95% CI) | *p* | Group | Time | Group×Time |
| Chair rise (s)^a^ | 63 | 42 | 17.7  (16.4-19.1) | 18.2  (16.6-20.0) | 16.3  (15.0-17.8) | 15.7  (14.1-17.6) | 1.04  (0.90-1.19) | 0.597 | 15.0  (13.6-16.6) | 19.2  (17.0-21.5) | 0.79  (0.67-0.92) | 0.002 | 0.162 | 0.004 | 0.002 |
| 8ft-up-and-go (s)^a^ | 63 | 41 | 13.2  (12.6-13.7) | 13.2  (12.6-14.0) | 12.4  (11.9-13.0) | 13.0  (12.1-13.8) | 0.96  (0.89-1.04) | 0.326 | 12.2  (11.5-12.9) | 13.6  (12.7-14.5) | 0.90  (0.82-0.98) | 0.016 | 0.086 | 0.182 | 0.094 |
| Stair climb (s)^a^ | 56 | 20 | 21.1  (19.4-23.0) | 21.2  (18.7-24.0) | 19.3  (17.6-21.2) | 25.7  (22.1-30.0) | 0.75  (0.62-0.90) | 0.002 | 18.9  (17.0-21.0) | 29.2  (24.8-34.3) | 0.65  (0.53-0.79) | 0.000 | 0.000 | 0.108 | 0.000 |
| Preferred gait  speed (m/s) | 63 | 41 | 0.75  (0.73-0.77) | 0.74  (0.71-0.77) | 0.77  (0.74-0.79) | 0.76  (0.72-0.79) | 0.01  (-0.03-0.06) | 0.642 | 0.79  (0.76-0.82) | 0.70  (0.66-0.74) | 0.09  (0.04-0.14) | 0.001 | 0.020 | 0.427 | 0.020 |
| Maximal gait  speed (m/s) | 63 | 41 | 1.04  (1.01-1.06) | 1.03  (0.99-1.06) | 1.08  (1.05-1.11) | 0.99  (0.95-1.03) | 0.09  (0.04-0.14) | 0.001 | 1.07  (1.03-1.11) | 0.98  (0.93-1.02) | 0.09  (0.03-0.16) | 0.003 | 0.000 | 0.856 | 0.020 |

Estimated means and 95% confidence intervals (95% CI) using linear mixed models. ^a^ Between-group differences for transformed variables are presented as ratio of the geometric mean for RTG to the geometric mean for CG and 95% CI.

RTG, Resistance training group; CG, Control group.
