# Supplementary material for: Effectiveness of a resistance training program on physical function, muscle strength, and body composition in community-dwelling older adults receiving home care: a cluster-randomized controlled trial
Source: Eur Rev Aging Phys Act. 2020 Aug 7;17:11. doi: 10.1186/s11556-020-00243-9 (PMC7414534; doi:10.1186/s11556-020-00243-9)
Supplement: Supplementary file 5 — Additional file 5:. Sensitivity analysis of stair climb, adjusted for vertical climb and number of steps. Values are estimated means and 95% confidence intervals (95% CI), unless stated otherwise. This additional file is a table (.docx) showing results from the sensitivity analysis of stair climb, where we adjusted for vertical climb in one model and number of steps in another model. [file 11556_2020_243_MOESM5_ESM.docx]

Table S5 Sensitivity analysis of stair climb, adjusted for vertical climb and number of steps.

| Outcome | Analyzed | | Baseline  Mean (95% CI) |  | 4 months | |  | Between-group  difference | |  | 8 months | |  | Between-group difference | |
| --- | --- | --- | --- | --- | --- | --- | --- | --- | --- | --- | --- | --- | --- | --- | --- |
|  | RTG  n | CG  n |  |  | RT  Mean (95% CI) | CG  Mean (95% CI) |  | Ratio (95% CI) | *p* |  | RT  Mean (95% CI) | CG  Mean (95% CI) |  | Ratio  ( 95% CI) | *p* |
| Stair climb (s)* | 56 | 20 | 26.4 (21.8-32.1) |  | 24.0 (19.6-29.4) | 32.2 (25.1-41.4) |  | 0.74 (0.61-0.91) | 0.004 |  | 23.3 (18.8-28.9) | 35.8 (27.6-46.5) |  | 0.65 (0.52-0.81) | 0.000 |
| Stair climb (s)** | 56 | 20 | 26.4 (21.7-32.0) |  | 24.0 (19.5-29.4) | 32.1 (25.0-41.4) |  | 0.75 (0.61-0.91) | 0.004 |  | 23.3 (18.8-28.8) | 35.7 (27.5-46.4) |  | 0.65 (0.52-0.81) | 0.000 |

Estimated means and 95% confidence intervals (95% CI) using linear mixed models. Between-group differences are presented as ratio of the geometric mean for RTG to the geometric mean for CG and 95% CI. * Adjusted for vertical climb. ** Adjusted for number of steps.

RTG, Resistance training group; CG, Control group.
